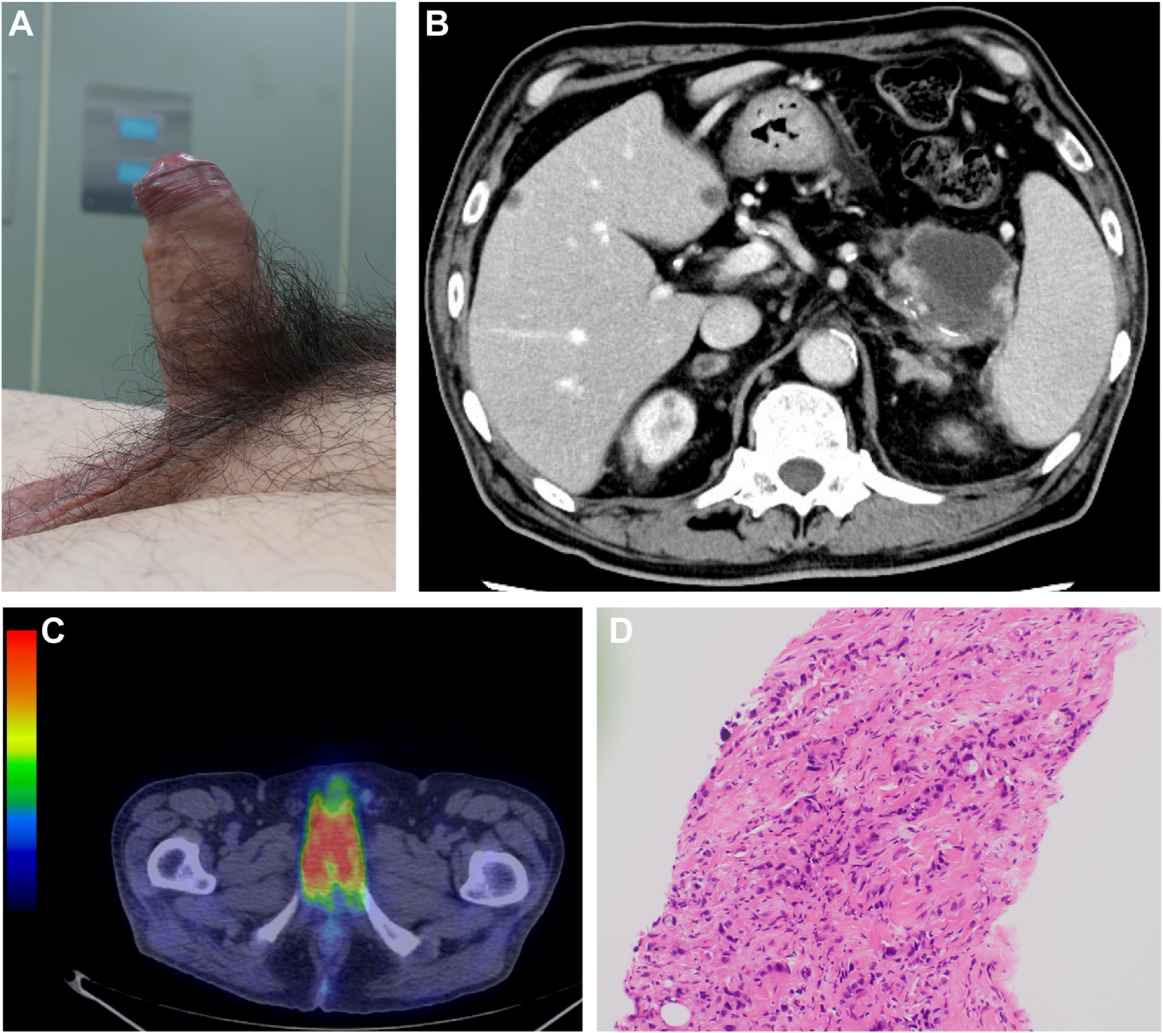# A Rare Cause of Priapism

**DOI:** 10.1016/j.gastha.2023.07.011

**Published:** 2023-07-20

**Authors:** Yujiro Kawakami, Ko Kobayashi, Hiroshi Nakase

**Affiliations:** 1 Department of Gastroenterology and Hepatology, Sapporo Medical University School of Medicine, Sapporo, Japan; 2 Department of Urology, Sapporo Medical University School of Medicine, Sapporo, Japan

A 68-year-old man who complained of epigastric pain, penile pain, and priapism was referred to our hospital (Figure A). Blood tests showed elevated serum carbohydrate antigen 19-9 (582 U/mL). Contrast-enhanced computed tomography revealed a 40-mm hypovascular tumor with a retention cyst at the pancreatic tail and multiple liver tumors, which was suggestive of pancreatic cancer with liver metastases (Figure B). We could not perform endoscopic ultrasonography-guided fine needle biopsy of the pancreatic tumor; thus, percutaneous needle biopsy of the liver lesion was performed. Pathological examination of the liver lesions revealed adenocarcinoma. Positron-emission tomography revealed increased ^18^F-fluorodeoxyglucose uptake in the penile region (Figure C). We performed corporoglanular shunt and needle biopsy of the penile lesion, and histopathology revealed adenocarcinoma similar to liver metastases (Figure D). Based on pathological findings, we diagnosed the patient with pancreatic adenocarcinoma with penile metastasis. He received chemotherapy with gemcitabine and nab-paclitaxel, however, died 4 months after starting chemotherapy.

Cancer metastasis to the penis is rare, and the main primary lesions are the bladder, prostate, gastrointestinal tract (mainly colorectal), and lung cancers. Penile metastases of pancreatic cancer are exceedingly rare. Patients with penile metastasis complain of severe penile pain, hematuria, and dysuria associated with abnormal and persistent erections.

**Figure:**